# Enhancing Ion Transport in Polymer Electrolytes by Regulating Solvation Structure via Hydrogen Bond Networks

**DOI:** 10.3390/molecules30112474

**Published:** 2025-06-05

**Authors:** Yuqing Gao, Yankui Mo, Shengguang Qi, Mianrui Li, Tongmei Ma, Li Du

**Affiliations:** Guangdong Provincial Key Laboratory of Fuel Cell Technology, School of Chemistry and Chemical Engineering, South China University of Technology, Guangzhou 510640, China

**Keywords:** polymer electrolyte, lithium–ion batteries, ion transport, H–bond network, solvation structures

## Abstract

Polymer electrolytes (PEs) provide enhanced safety for high–energy–density lithium metal batteries (LMBs), yet their practical application is hampered by intrinsically low ionic conductivity and insufficient electrochemical stability, primarily stemming from suboptimal Li^+^ solvation environments and transport pathways coupled with slow polymer dynamics. Herein, we demonstrate a molecular design strategy to overcome these limitations by regulating the Li^+^ solvation structure through the synergistic interplay of conventional Lewis acid–base coordination and engineered hydrogen bond (H–bond) networks, achieved by incorporating specific H–bond donor functionalities (N,N′–methylenebis(acrylamide), MBA) into the polymer architecture. Computational modeling confirms that the introduced H–bonds effectively modulate the Li^+^ coordination environment, promote salt dissociation, and create favorable pathways for faster ion transport decoupled from polymer chain motion. Experimentally, the resultant polymer electrolyte (MFE, based on MBA) enables exceptionally stable Li metal cycling in symmetric cells (>4000 h at 0.1 mA cm^−2^), endows LFP|MFE|Li cells with long–term stability, achieving 81.0% capacity retention after 1400 cycles, and confers NCM622|MFE|Li cells with cycling endurance, maintaining 81.0% capacity retention after 800 cycles under a high voltage of 4.3 V at room temperature. This study underscores a potent molecular engineering strategy, leveraging synergistic hydrogen bonding and Lewis acid–base interactions to rationally tailor the Li^+^ solvation structure and unlock efficient ion transport in polymer electrolytes, paving a promising path towards high–performance solid–state lithium metal batteries.

## 1. Introduction

The rapid proliferation of electric vehicles and large–scale energy storage systems has intensified the demand for lithium–ion batteries (LIBs) with significantly improved energy density and safety profiles [1,2,3]. Lithium metal batteries (LMBs), which utilize lithium metal anodes with an ultrahigh theoretical capacity, are widely regarded as promising candidates for next–generation energy storage technologies. However, their practical deployment is impeded by critical challenges–foremost among them the electrolyte, which governs ion transport and plays a decisive role in determining battery safety, electrochemical stability, and cycling performance. Conventional liquid electrolytes (LEs), although widely adopted, suffer from inherent drawbacks such as flammability, leakage, and limited electrochemical stability–particularly under high–voltage conditions–which restrict their suitability for high–energy–density applications [4,5].

Polymer electrolytes (PEs) have emerged as a promising alternative to LEs, offering intrinsic advantages including non–volatility, mechanical flexibility, and enhanced safety [6,7,8]. Nevertheless, most conventional PEs, especially those based on poly(ethylene oxide) (PEO), exhibit low ionic conductivity at room temperature (<10^−5^ S cm^−1^) and insufficient oxidative stability. These dual limitations critically hinder their ability to satisfy the simultaneous demands of high energy density and high power output, constituting a critical bottleneck for the commercialization of PEs. The ion transport in traditional PEs is typically coupled with polymer segmental dynamics, which are intrinsically sluggish at room temperature. Although plasticizer incorporation can enhance ionic conductivity, excessive amounts often compromise the mechanical robustness and thermal stability of the electrolyte. Moreover, the presence of plasticizers may lead to inhomogeneous ion distribution across the electrolyte and electrode/electrolyte interfaces, potentially triggering lithium dendrite formation and exacerbating interfacial side reactions [9,10,11]. In addition, the limited oxidative stability of both the polymer matrix and common plasticizers constrains the accessible voltage window of the battery system [12,13,14,15].

**Figure 1:**
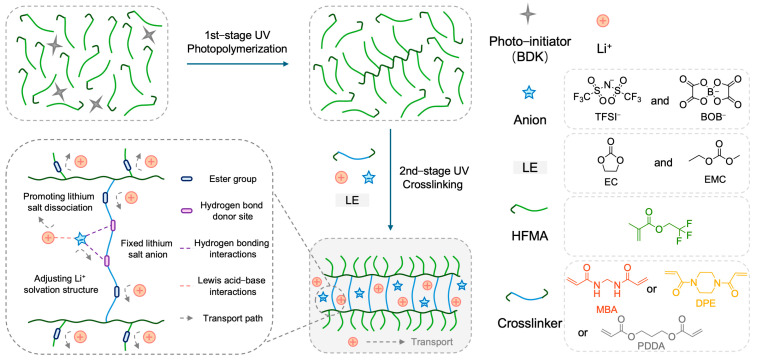
Chemical structures of monomers and crosslinked polymer networks used in MFE, DFE, and PFE.

Fundamentally, ion solvation and transport behaviors in electrolytes are governed by Lewis acid–base interactions. Lithium ions (Li^+^), functioning as Lewis acids, preferentially coordinate with Lewis base sites–atoms or functional groups possessing lone pairs–within the electrolyte framework [16,17]. The strength and configuration of these interactions profoundly affect salt dissociation, the structure of the solvation sheath, and ultimately, ionic mobility. Recent advances have shown that incorporating hydrogen–bond (H–bond) networks can decouple ion transport from polymer segmental motion and solvent volatility, thereby enabling rapid conduction while maintaining electrochemical integrity [18,19,20]. Notably, diverse H–bond donor groups–such as hydroxyl (–OH) and amide (–NH) functionalities–have been explored [21]. Amide–based donors in particular impart self–healing and strong adhesive properties, enhancing interphase compatibility, mechanical robustness, and safety in polymer electrolytes. For example, a waterborne polyurethane crosslinked via amide H–bonds exhibited a tensile strength of 0.9 MPa and elongation at break of 140%, but its ionic conductivity at room temperature remained limited to 10^−5^ S cm^−1^, highlighting the trade–off between mechanical strength and ion transport [22]. While H–bond networks can greatly improve mechanical performance, improperly designed networks may hinder ionic conductivity and electrochemical stability. Our previous work demonstrated that a judiciously engineered H–bond network not only enhances polymer electrolyte stability but also promotes Li^+^ transport [23], and that the rational molecular design of non–covalent interactions can further optimize electrolyte properties [24]. However, the molecular–level mechanism by which H–bond networks mediate Li^+^ transport remains unclear. Consequently, a key challenge is the rational design of polymer architectures that leverage synergistic Lewis acid–base coordination and hydrogen bonding (Figure 1) to simultaneously boost ion transport kinetics and electrochemical stability in polymer electrolytes.

Herein, we propose a polymer electrolyte design strategy centered on regulating the Li^+^ solvation structure through the synergistic interplay of Lewis acid–base coordination and hydrogen bonding interactions. By incorporating specific H–bond donor motifs into the polymer backbone alongside Lewis base sites, we aim to modulate the local environment around Li^+^, thereby facilitating more efficient ion transport pathways. Computational investigations employing Density Functional Theory (DFT) and Molecular Dynamics (MD) simulations elucidated the pivotal role of H–bonding in modulating ion transport properties. Our findings reveal that the rationally introduced H–bond networks effectively promote lithium salt dissociation and accelerate ion transport. Concurrently, these interactions contribute to a more uniform distribution of Li^+^ ions throughout the electrolyte and stabilize the electrode–electrolyte interface, enabling robust electrochemical performance. The practical viability and effectiveness of this molecular design approach were subsequently demonstrated through comprehensive electrochemical evaluations of the resulting polymer electrolyte (denoted MFE), showcasing its capability to support stable operation in various battery configurations, including assessments with lithium metal anodes and high–voltage cathodes. This work highlights a molecular–level strategy to harness synergistic H–bonding and Lewis acid–base interactions for solvation structure regulation and enhanced ion transport, offering a promising route toward the development of high–performance, high–energy–density LIBs.

## 2. Results and Discussion

### 2.1. Design, Preparation, and Structural Characterization of Polymer Electrolytes

The rational design of the polymer network architecture is paramount for modulating ion transport properties within polymer electrolytes (PEs). This study aimed to enhance Li^+^ transport by constructing a crosslinked network incorporating hydrogen–bond–capable amide functionalities, synergistically combined with plasticizers. Figure 1 illustrates the chemical structures of the key monomers: N,N′–methylenebis(acrylamide) (MBA) as the primary bifunctional crosslinker bearing amide groups, 2,2,2–trifluoroethyl methacrylate (HFMA) as a fluorinated monomer, and comparative crosslinkers 1,4–diacryloylpiperazine (DPE) and propane–1,3–diyl diacrylate (PDDA). Utilizing these components alongside EC/EMC plasticizers and a LiTFSI/LiBOB dual–salt system, three distinct PEs (MFE, DFE, and PFE, corresponding to MBA, DPE, and PDDA crosslinkers, respectively) were synthesized via our previously reported two–stage UV–induced copolymerization strategy [23,24]. This strategy is based on the viscosity increase in the partially polymerized HFMA monomer during the first stage of polymerization, as schematically depicted in Figure 1. The selection of DPE and PDDA, which differ significantly from their corresponding MBA–crosslinked networks in polarity and hydrogen–bonding capability, provides a basis for evaluating the impact of network structure on electrolyte performance.

**Figure 2:**
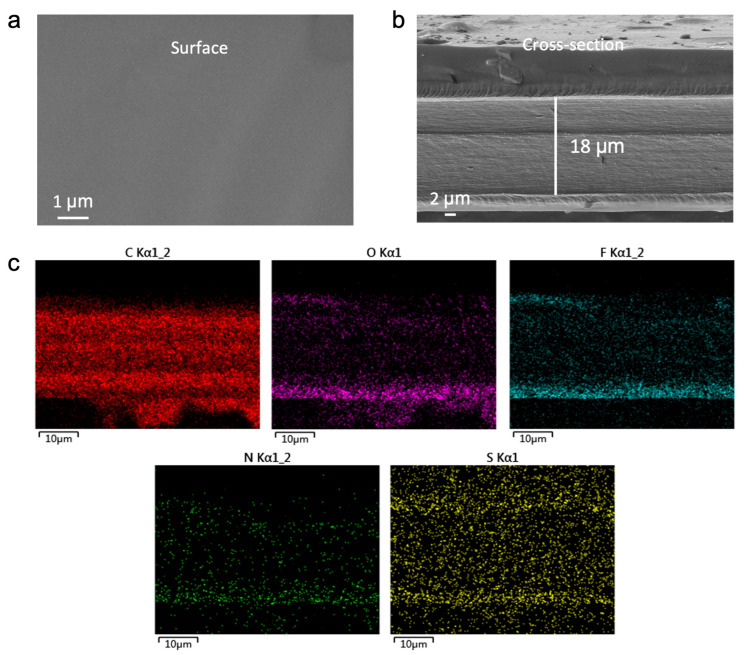
(**a**) Surface and (**b**) cross–sectional SEM images of the MFE membrane. (**c**) Elemental EDS mapping of C, O, F, N, and S.

To ascertain the morphological integrity and compositional homogeneity of the fabricated electrolytes, the representative MFE membrane was characterized using scanning electron microscopy (SEM) and energy–dispersive X–ray spectroscopy (EDS). As revealed by the top–view SEM image (Figure 2a), the MFE membrane exhibits a smooth, dense, and largely defect–free surface, indicative of uniform film formation via the UV polymerization process. Cross–sectional SEM analysis (Figure 2b) corroborates this uniformity, showing a well–defined polymer layer with a thickness of approximately 18 µm. The compact structure and absence of discernible phase separation between the polymer network and the embedded liquid components attest to successful crosslinking and formulation homogeneity.

EDS elemental mapping (Figure 2c) provided further insight into the spatial distribution of key constituents within the MFE membrane. The maps demonstrate a homogeneous dispersion of carbon (C), oxygen (O), fluorine (F), nitrogen (N), and sulfur (S) elements throughout the matrix. Particularly noteworthy is the uniform distribution of N (originating from the MBA crosslinker) and F (from the HFMA monomer), confirming their successful and even incorporation into the polymer network. The consistent S signal, primarily attributed to the TFSI^−^ anions, indicates effective dissolution and dispersion of the lithium salts. Collectively, these SEM and EDS findings validate the excellent structural integrity and compositional uniformity of the MFE electrolyte, establishing critical prerequisites for consistent ion conduction and the formation of continuous ion transport pathways.

### 2.2. Theoretical Insights: Molecular Interactions, Coordination Environment, and Electronic Structure

**Figure 3:**
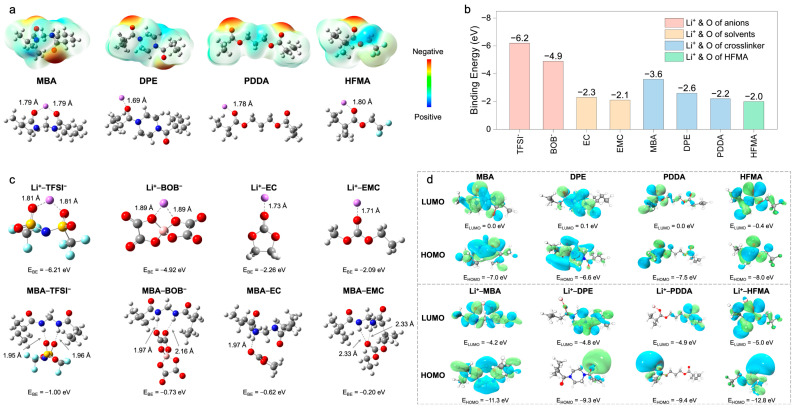
(**a**) ESP distributions and coordination geometries with Li^+^ of MBA, DPE, PDDA, and HFMA. (**b**) Binding energies of Li^+^ with various ligands. (**c**) Binding configurations and interaction energies between Li^+^ and electrolyte components, and between MBA and these components via hydrogen bonding. (**d**) HOMO and LUMO of free monomers, and energy levels after Li^+^ coordination.

To fundamentally elucidate the mechanisms underpinning enhanced ion transport, Density Functional Theory (DFT) calculations and Molecular Dynamics (MD) simulations were performed. These computational approaches provide atomistic– and molecular–level insights into the intricate interplay of intermolecular interactions, electronic structures, and the resultant Li^+^ coordination environments within the polymer electrolytes (PEs), with a specific focus on delineating the structural influence exerted by different crosslinkers. The initial DFT investigations centered on the electrostatic potential (ESP) distributions of the constituent monomers and their subsequent coordination behavior with Li^+^ (Figure 3a). The ESP map computed for N,N*′*–methylenebis(acrylamide) (MBA) prominently features pronounced negative–potential zones localized around the amide carbonyl oxygen atoms. This characteristic signifies a strong electron–donating propensity, thereby facilitating potent, bidentate coordination with Li^+^, as evidenced by the calculated short Li–O bond distance of approximately 1.79 Å. In stark contrast, 1,6–diisocyanatohexane–based polyurea (DPE) exhibits a more diffuse ESP landscape, translating into weaker Li^+^ coordination (Li–O = 1.69 Å). Poly(ethylene glycol) diacrylate (PDDA) demonstrates intermediate coordination strength (Li–O = 1.78 Å), while hexafluorobutyl methacrylate (HFMA), despite possessing polar ester functionalities, presents a comparatively lower surface electron density and, consequently, a diminished affinity for Li^+^ (Li–O = 1.80 Å). This computational evidence collectively suggests that the bifunctional amide groups intrinsic to the MBA structure furnish the most potent and localized coordination sites for Li^+^, a feature anticipated to be highly conducive to promoting lithium salt dissociation.

**Figure 4:**
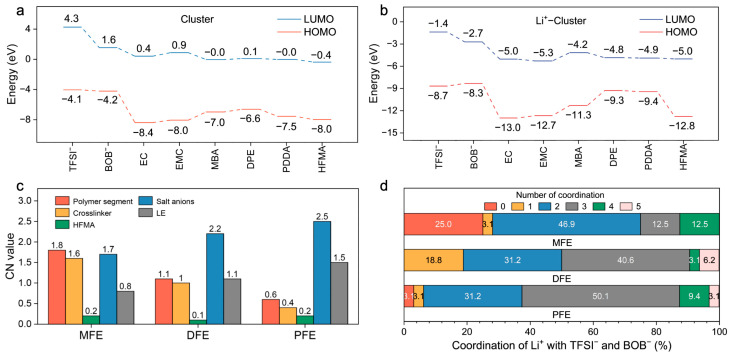
(**a**) Frontier orbital energies of electrolyte components. (**b**) Energy shifts upon Li^+^ coordination. (**c**) Average CNs of Li^+^ with various components. (**d**) CN distribution with anions.

A further quantitative assessment of interaction strengths was performed through binding energy ($E_{BE}$) calculations between Li^+^ and various electrolyte constituents (Figure 3b,c). The computations reveal dominant electrostatic attractions governing Li^+^–anion interactions (Li^+^–TFSI^−^: −6.2 eV; Li^+^–BOB^−^: −4.9 eV), substantially exceeding those observed for Li^+^–plasticizer pairings (Li^+^–EC: −2.3 eV; Li^+^–EMC: −2.1 eV) (Figure 3b). Although the corresponding short Li–O bond lengths (1.71–1.89 Å, Figure 3c) corroborate intimate coordination, these potent Li^+^–anion associations intrinsically foster the formation of ion aggregates, which detrimentally impede Li^+^ mobility. To counteract this unfavorable ion pairing, the MBA crosslinker was strategically incorporated. The DFT calculations confirm that MBA possesses a notably higher preferential binding affinity for Li^+^ (−3.6 eV) relative to DPE (−2.6 eV), PDDA (−2.2 eV), and HFMA (−2.0 eV) (Figure 3b), an advantage primarily attributed to its dual amide configuration. Critically, beyond direct coordination, MBA actively modulates the ionic microenvironment via hydrogen bonding networks (Figure 3c). It establishes hydrogen bonds with anions (characteristic distances ~1.95–2.16 Å), thereby disrupting direct Li^+^–anion coordination shells, and concurrently interacts with solvent molecules (e.g., MBA–EC $E_{BE}$: −0.52 eV, distance: ~2.33 Å), influencing the overall Li^+^ solvation sheath structure. Consequently, MBA operates synergistically not merely as a passive structural crosslinker but as an active modulator of the Li^+^ coordination sphere, leveraging both direct Lewis acid–base coordination and strategically engineered hydrogen bond interactions to facilitate salt dissociation and restructure the local ionic environment. A complementary analysis of frontier molecular orbitals (HOMO/LUMO) was conducted to evaluate the intrinsic electronic stability (Figure 3d and Figure 4a). All monomers exhibit relatively low HOMO energy levels (−6.6 to −8.0 eV), indicative of robust oxidative stability (Figure 4a). Upon coordination with Li^+^, general stabilization (lowering) of all component orbital energy levels is observed, effectively widening the electrochemical stability window of the electrolyte system (Figure 4b). Notably, the Li^+^–MBA complex displays a significantly stabilized HOMO level (−11.3 eV) and the lowest LUMO level (−4.2 eV) among the investigated crosslinker complexes, predicting superior electrochemical resilience against both oxidative and reductive degradation, a finding consistent with its strong Li^+^ interaction propensity.

Complementing the static DFT insights, the MD simulations furnished dynamic perspectives on the Li^+^ solvation structure, primarily through the analysis of radial distribution functions (RDFs) and coordination numbers (CNs) (Figure S1a–h and Figure 4c,d). An examination of the Li^+^–polymer RDF profiles (Figure S1a) reveals the most pronounced peak intensity and, consequently, the highest CN, within the MFE system, with a particular contribution from the MBA units (Figure S1c). This observation signifies a strong, preferential interaction between Li^+^ and the MFE polymer backbone. Conversely, the Li^+^–anion RDFs (Figure S1b,e,f) demonstrate markedly attenuated coordination intensity in the MFE system compared to the DFE and PFE analogs. This finding corroborates the hypothesis that robust Li^+^–polymer/solvent interactions, facilitated by the MBA crosslinker in MFE, effectively compete against and diminish direct Li^+^–anion pairing. Furthermore, MFE exhibited slightly decreased Li^+^–solvent interactions (Figure S1g) and reduced propensity for Li^+^–Li^+^ aggregation (Figure S1h). Quantitative CN analysis (Figure 4c,d) substantiates these structural observations: Li^+^ ions within the MFE network preferentially coordinate with polymer segments (especially MBA moieties), concurrently exhibiting the lowest average CNs with anions and solvent molecules among the systems studied. The distribution of Li^+^–anion CNs (Figure 4d) further underscores this trend; approximately 75% of Li^+^ ions in MFE coordinate with two or fewer anions, whereas this percentage markedly decreases in DFE (50.1%) and PFE (37.4%). Collectively, these MD simulation results unequivocally demonstrate that the MBA–based MFE network architecturally fosters a Li^+^ solvation environment substantially more conducive to ion transport by promoting Li^+^–polymer interactions while simultaneously suppressing deleterious Li^+^–anion associations. Studies have demonstrated that the distribution and solvation structure of lithium ions significantly affect ion transport behavior [25,26]. Further Mean Squared Displacement (MSD) analysis based on MD simulations (Figure S2) reveals that the MFE–based polymer electrolyte exhibits a higher Li^+^ diffusion coefficient (1.9 × 10^−5^ cm^2^ s^−1^). This value substantially exceeds those calculated for the DFE (1.6 × 10^−5^ cm^2^ s^−1^) and PFE (1.5 × 10^−5^ cm^2^ s^−1^) systems, underscoring the enhanced ion transport dynamics within the MFE matrix. Finally, an analysis of component spatial density profiles derived from the MD simulations was employed to assess the microstructural homogeneity of the systems (Figure S3a–d). The MFE system displayed conspicuously higher and more uniform density distributions for Li^+^ ions (Figure S3a), polymer ester oxygens (Figure S3b), and anion–representative atoms (S, B; Figure S3c–d) throughout the simulation box. In contrast, both DFE and PFE exhibited lower overall densities and more pronounced spatial fluctuations, indicative of less homogeneous microstructures. This comparative analysis strongly suggests that the engineered network incorporating MBA promotes a highly uniform electrochemical environment, likely facilitating the formation of continuous and efficient ion conduction pathways, thereby rationalizing the experimentally observed enhancements in ionic conductivity.

### 2.3. Electrochemical Performance Evaluation

**Figure 5:**
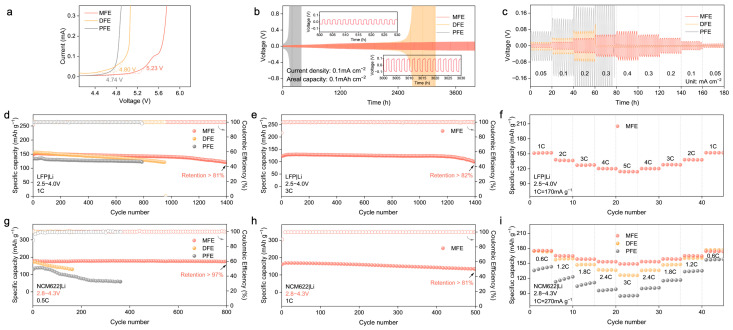
(**a**) LSV curves of SS|PEs|Li cells scanned from 3.0 V to 6.0 V. (**b**) Long–term cycling of Li|PEs|Li cells. (**c**) Performance under varied current densities. (**d**) Cycling of LFP|Li cells at 1C, with an areal cathode capacity of ~0.37 mAh·cm^−2^. (**e**) Cycling at 3C, with an areal cathode capacity of ~0.32 mAh·cm^−2^. (**f**) Rate capability of LFP|Li cell using MFE at various C–rates. (**g**) Cycling performance of NCM622|Li cells with MFE, DFE, and PFE electrolytes at 0.5C (2.8–4.3 V), with an areal cathode capacity of ~0.45 mAh·cm^−2^. (**h**) Cycling performance of NCM622|Li cell using MFE electrolyte at 1C (2.8–4.3 V), with an areal cathode capacity of ~0.52 mAh·cm^−2^. (**i**) Rate capability comparison of NCM622|Li cells using MFE, DFE, and PFE. All full cells were cycled under symmetric charge/discharge rates.

Building upon the structural insights and theoretical predictions highlighting the advantages conferred by the MBA–crosslinked network, the electrochemical performance of the synthesized polymer electrolytes (MFE, DFE, and PFE) was systematically interrogated. Linear sweep voltammetry (LSV) was employed to probe the oxidative stability limits (Figure 5a and Figure S4). The MFE electrolyte exhibited markedly enhanced oxidative stability, with electrochemical decomposition commencing at approximately 5.23 V vs. Li/Li^+^. This threshold considerably surpasses those observed for DFE (~4.80 V) and PFE (~4.74 V). This expanded electrochemical window, consistent with the DFT–predicted low HOMO energy level and the stabilized Li^+^–MBA complex, affirms MFE’s intrinsic compatibility with high–voltage cathode materials. Subsequently, the interfacial stability and compatibility with lithium metal anodes were investigated via galvanostatic cycling of Li|PEs|Li symmetric cells (Figure 5b,c). The MFE–based cell demonstrated exceptional long–term cycling stability, maintaining operation for over 4000 h at a current density of 0.1 mA cm^−2^ with remarkably low and stable voltage polarization (Figure 5b). This exceptional stability underscores the formation of a highly robust and uniform solid electrolyte interphase (SEI) on the lithium metal anode and testifies to the effective suppression of Li dendrite proliferation facilitated by the MFE structure. In stark contrast, cells utilizing DFE and PFE electrolytes experienced premature failure under identical conditions. The Li|Cu half–cell configuration was used to evaluate the reversibility of lithium plating/stripping in different polymer electrolyte systems via cyclic voltammetry (Figure S5). Among the three systems, the MFE–based electrolyte exhibited the highest peak current density and most symmetric redox behavior, indicative of highly reversible lithium deposition and stable interfacial kinetics. In contrast, the PFE system showed the lowest current response and pronounced polarization, reflecting poor interfacial compatibility and limited electrochemical reversibility. These results corroborate the superior ion transport and interfacial characteristics of the MFE electrolyte, which can be attributed to its hydrogen–bond–regulated solvation architecture. Furthermore, under incrementally increasing current densities (Figure 5c), the MFE–based cell consistently sustained the lowest overpotential, underscoring its capacity for stable lithium deposition/stripping processes even at elevated rates.

**Figure 6:**
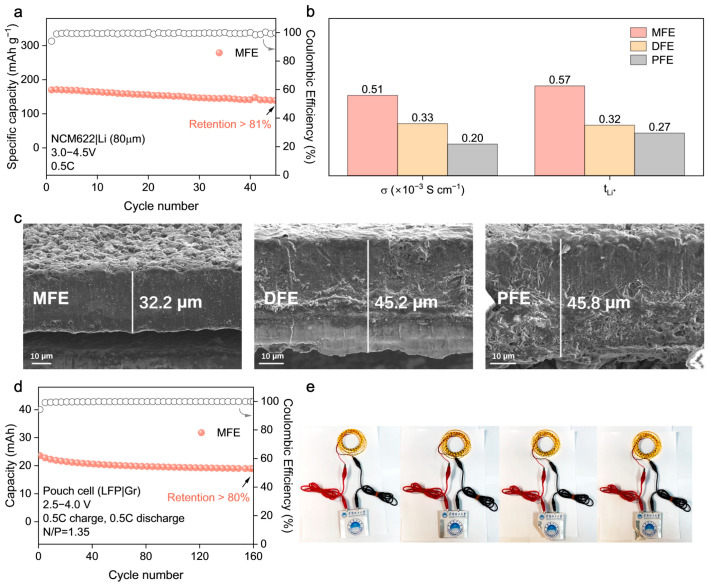
(**a**) Cycling of NCM622|Li cells with high mass loading (~1.36 mAh·cm^−2^) under a 3.0–4.5 V voltage window. (**b**) Comparison of σ and t_Li+_ in different electrolytes. (**c**) SEM of Li anodes after 200 cycles with MFE, DFE, and PFE. (**d**) Cycling performance of LFP|Gr pouch cell using MFE. (**e**) Photographs of MFE–powered pouch cell lighting an LED light strip. All full cells were cycled with symmetric charge/discharge rates.

The practical viability of the MFE electrolyte was further scrutinized in lithium cell configurations employing both LiFePO_4_ (LFP) and LiNi_0.6_Co_0.2_Mn_0.2_O_2_ (NCM622) cathodes against lithium metal anodes. Within LFP|Li cells (Figure 5d–f), MFE considerably surpassed the control electrolytes, delivering remarkable long–term cyclability: retaining over 81% of its initial capacity after 1400 cycles at 1C (Figure 5d). The corresponding evolution of voltage profiles for LFP|Li full cells at the 100th, 200th, and 400th cycles (Figure S6) further highlighted the minimal capacity decay achieved with the MFE. Impressively, even under a more demanding 3C rate protocol, the MFE–based cell maintained 82% capacity retention after 1400 cycles (Figure 5e). Moreover, the initial electrochemical impedance spectroscopy (EIS) measurements (Figure S7) of LFP|Li full cells assembled with the MFE exhibited the lowest interfacial impedance, signifying superior interfacial kinetics and reduced resistance to ion transport. Rate capability assessments (Figure 5f) further corroborated the superior kinetic performance enabled by MFE, which delivered a substantial capacity of approximately 120 mAh g^−1^ at a high rate of 5C, alongside excellent capacity recovery upon returning to lower rates. Transitioning to higher–voltage NCM622|Li cells operating between 2.8 and 4.3 V (Figure 5g–i), MFE demonstrated similarly superior electrochemical characteristics. At a rate of 0.5C, the MFE cell retained over 97% of its initial discharge capacity after 800 cycles accompanied by near–unity CE, starkly contrasting with the significant capacity decay observed in control cells employing DFE and PFE electrolytes (Figure 5g and Figure S8). At an elevated rate of 1C, the MFE cell sustained stable cycling, retaining over 81% capacity after 500 cycles (Figure 5h). Correspondingly, rate capability evaluations (Figure 5i) revealed that MFE consistently delivered higher specific capacities across a range of rates from 0.6C to 3C, affirming its capability to facilitate rapid ion transport even under the demanding conditions imposed by the high–voltage cathode. Notably, when benchmarked against previous research on polymer electrolytes (PEs), our MFE system demonstrates superior long–term stability and excellent compatibility with high–voltage cathode materials such as NCM622 (Table S1). Critically, the robustness of the MFE electrolyte was confirmed under more challenging practical conditions, including a high cathode mass loading (~4 mg cm^−2^) and an extended voltage window (3.0–4.5 V), where it maintained over 81% capacity retention after 45 cycles at 0.5C (Figure 6a), highlighting its potential for practical applications.

The origin of MFE’s superior electrochemical performance is elucidated by key ion transport parameters (Figure 6b, Figures S9 and S10; Table S2), which provide a clear rationale for its advantageous behavior. MFE distinctly exhibits the highest room–temperature ionic conductivity among the synthesized electrolytes (σ ≈ 5.1 × 10^−4^ S cm^−1^) alongside a remarkably enhanced Li^+^ transference number ($t_{{Li}^{+}}$ ≈ 0.57). These values substantially surpass those measured for DFE ($t_{{Li}^{+}}$ ≈ 0.32) and PFE ($t_{{Li}^{+}}$ ≈ 0.27). This substantially elevated $t_{{Li}^{+}}$, corroborated by the computationally predicted reduction in Li^+^–anion coordination within the MFE system, confirms that Li^+^ migration dominates charge transport. This characteristic is pivotal as it minimizes the formation of concentration polarization gradients during battery operation, thereby contributing significantly to the observed high–rate capability and exceptional cycling stability previously described. Beyond the quantitative transport parameters, the underlying structural origin of the enhanced ion conduction in the MFE system can be further understood by considering the role of hydrogen bonding interactions within the cross–linked polymer network. In particular, both intra–polymer and inter–polymer hydrogen bonds are likely to coexist and synergistically influence ion transport behavior. The bifunctional crosslinker MBA introduces amide groups (–NH–) that serve as hydrogen bond donors. Intra–polymer hydrogen bonds formed within individual chains enhance local rigidity and prevent segmental collapse, preserving favorable polymer chain conformations for Li^+^ migration. Meanwhile, inter–polymer hydrogen bonding between adjacent chains contributes to the formation of a dynamic three–dimensional network that promotes both mechanical robustness and ion–conductive continuity. More critically, these hydrogen bonding motifs can selectively interact with the anions of lithium salts, immobilizing them via directional hydrogen bonding and thereby facilitating salt dissociation. This mechanism reduces ion pairing and enhances the effective lithium–ion concentration, which rationalizes the observed improvement in Li^+^ transference number and ionic conductivity in the MFE system. Taken together, the dual–mode hydrogen bonding network plays a pivotal role in modulating the solvation environment and constructing efficient ion transport pathways within the polymer matrix.

### 2.4. Interfacial Stability Analysis and Pouch Cell Validation

Given that the nature of the electrode–electrolyte interphase is a critical determinant of cycling longevity and safety in lithium metal batteries, the morphology of lithium metal anodes retrieved after prolonged cycling (200 cycles in NCM622|PEs|Li cells) was meticulously inspected using Scanning Electron Microscopy (SEM) (Figure 6c). The anode surface exposed to the MFE electrolyte exhibited a notably smooth and compact deposition morphology, largely devoid of deleterious dendritic protrusions or significant porosity. This favorable morphology strongly indicates the formation of a stable, homogeneous solid electrolyte interphase (SEI) facilitated by the MFE network, which effectively mitigates detrimental localized current fluctuations and regulates uniform lithium deposition/stripping. Conversely, lithium anodes cycled with the DFE and PFE electrolytes displayed pronounced surface roughness characterized by non–uniform deposition patterns and evidence of incipient dendrite formation, observations consistent with, and providing microstructural corroboration for, their previously documented inferior electrochemical cycling stability and higher interfacial impedance.

To further investigate the mechanical properties of the polymer electrolytes (PEs), Atomic Force Microscopy (AFM) was employed, with the results detailed in Figures S11 and S12. The AFM topography images (Figure S11) were first analyzed to assess surface roughness. The MFE membrane exhibited the lowest surface roughness (R_q_ = 16.7 nm, R_a_ = 13.6 nm), indicative of a dense and uniform polymer network facilitated by MBA–mediated cross–linking and hydrogen bond reinforcement. In contrast, the PFE membrane displayed a significantly rougher surface (R_q_ = 62.4 nm), suggesting inferior structural uniformity and potentially compromised interfacial compatibility. These observations are consistent with the SEM results presented in Figure 2. Subsequently, the nanomechanical properties of the polymer electrolyte membranes were evaluated using AFM–based quantitative nanomechanical mapping (QNM), as depicted in Figure S12. Among the three systems, the MFE membrane demonstrated the highest average Young’s modulus (239 MPa) with a remarkably uniform spatial distribution. This is characteristic of a well–organized and highly crosslinked polymer network, further reinforced by hydrogen bonding. Conversely, the DFE sample showed the lowest modulus (118 MPa) and significant heterogeneity, reflecting a less cohesive structure with potential soft domains. The PFE membrane presented an intermediate Young’s modulus (224 MPa), albeit with greater nanoscale variation, suggesting localized rigidity but reduced overall structural uniformity. These findings underscore the critical role of molecular–level interactions in tailoring the mechanical robustness of PEs. Such robustness is essential for ensuring stable interfacial contact and effectively suppressing dendrite growth in LIBs.

To evaluate the practical applicability and potential for scalability of the optimized MFE system, prototype LFP|graphite (Gr) pouch cells were constructed and subjected to rigorous electrochemical testing (Figure 6d and Figure S14). These pouch cells demonstrated robust and stable cycling performance, retaining over 80% of their initial discharge capacity after 160 cycles under a 0.5C rate. This successful validation in a relevant device format not only confirms MFE’s electrochemical efficacy but also attests to its compatibility with commercially prevalent electrode materials (LFP, graphite) sufficient for practical cell assembly. The functional viability was further underscored by the demonstration of a fabricated pouch cell powering an array of light–emitting diodes (LEDs) (Figure 6e), visually confirming its potential for tangible energy storage applications.

## 3. Materials and Methods

### 3.1. Density Functional Theory (DFT) Calculations

First–principles DFT calculations were performed using the Gaussian 09 software package to elucidate intermolecular/ionic interactions, electronic structures, and Li^+^ coordination environments. The geometries of all species (crosslinkers MBA, DPE, PDDA; monomer HFMA; plasticizers EC and EMC; Li^+^, TFSI^−^, BOB^−^ ions; and related complexes) were optimized using the B3LYP hybrid functional with the def2TZVP basis set. Grimme’s D3 empirical dispersion correction was included. Frequency calculations confirmed all optimized structures as stable points (no imaginary frequencies). Based on the optimized geometries, the binding energies ($E_{\begin{array}{c} BE \end{array}}=E_{complex}-\sum E_{monomers}$, with BSSE correction), electrostatic potential (ESP, analyzed using Multiwfn and visualized with VMD), frontier molecular orbital energies (HOMO/LUMO for components and Li^+^–ligand clusters), and bond lengths (specifically Li–O in coordination clusters) were calculated and analyzed to provide theoretical insights into interaction strengths, reactivity sites, electrochemical stability, and Li^+^ solvation structures.

### 3.2. Molecular Dynamics (MD) Simulations

MD simulations were employed using the LAMMPS 2020 software package to investigate the influence of different crosslinkers (MBA, DPE, PDDA) on polymer electrolyte microstructure and ion transport. Interatomic interactions were described using a combined GAFF+UFF force field. The initial configurations and RESP charges for polymerized fragments (crosslinkers, HFMA), plasticizers (EC, EMC), Li^+^ ions, and anions (TFSI^−^, BOB^−^) were obtained as described previously (Section 3.1, B3LYP/def2–tzvp optimization). Three independent systems, differing only in crosslinker type while maintaining identical compositions for all other components (HFMA host, LiTFSI/LiBOB salts, EC/EMC plasticizers), were constructed using Materials Visualizer with periodic boundary conditions. The simulation protocol included energy minimization, NPT equilibration (until density/energy convergence), and a ≥10 ns NVT production run. The trajectory analysis focused on radial distribution functions (g(r)) for Li^+^ coordination structure, coordination numbers (CN) and ligand contributions (via RDF integration), and component density distributions to assess system homogeneity and crosslinker–specific effects on Li^+^ solvation and microstructure.

### 3.3. Polymer Electrolyte Preparation

The polymer electrolytes (PEs) were synthesized via a two–stage UV photopolymerization process. The materials used included N,N′–methylenebis(acrylamide) (MBA, 97%, Bide Pharmatech), 1,4–diacryloylpiperazine (DPE, 95%, Bide Pharmatech), propane–1,3–diyl diacrylate (PDDA, >95%, Bide Pharmatech), 2,2,2–trifluoroethyl methacrylate (HFMA, 98%, Bide Pharmatech), 2,2–dimethoxy–2–phenylacetophenone (BDK, Shanghai Yinchang New Materials), ethylene carbonate (EC, >99%, Macklin), ethyl methyl carbonate (EMC, 98%, Aladdin), lithium bis(trifluoromethanesulfonyl)imide (LiTFSI, 99.9%, Macklin), and lithium bis(oxalato)borate (LiBOB, ≥99%, Aladdin). Three PEs (MFE based on MBA, DFE based on DPE, PFE based on PDDA) were prepared (Figure 1, Table S3).

#### 3.3.1. Pre–Polymer Syrup (PPS) Preparation–First–Stage UV Photopolymerization

HFMA monomer (50 g) and the photo–initiator BDK (0.015 g) were added to a nitrogen–purged flask and stirred until a homogeneous solution was obtained. The mixture was then exposed to a 365 nm UV lamp for approximately 2 min. During irradiation, a noticeable increase in viscosity and the appearance of the rod–climbing phenomenon indicated the successful formation of a pre–polymer syrup (PPS) via partial photopolymerization. The reaction was carried out under a nitrogen atmosphere to prevent premature termination. Upon completion, the reaction was quenched by introducing air into the system, utilizing oxygen–inhibited radical polymerization to effectively terminate the reaction.

The polymerization process was further confirmed by FTIR spectroscopy (Figure S15). Specifically, the C=C stretching vibration peak at 1638 cm^−1^ decreased in intensity relative to the C=O stretching band at 1758 cm^−1^ after UV exposure, confirming the consumption of vinyl groups via radical–induced C–C bond formation. Notably, the persistence of a weak C=C band in the spectrum of the pre–polymerized sample indicates the presence of residual HFMA monomer, suggesting that the PPS comprises predominantly short–chain oligomers with trace amounts of unreacted monomer. This observation aligns well with the previously reported photopolymerization behavior of acrylate monomers [27,28]. The detailed kinetics and mechanisms of this first–stage UV curing step have been discussed in our prior work [23,29].

#### 3.3.2. Polymer Electrolyte Fabrication–Second–Stage UV Crosslinking

*Liquid Electrolyte (LE) Preparation:* A binary lithium salt system comprising LiTFSI (0.6 M) and LiBOB (0.4 M) was dissolved in an EC:EMC mixture (volume ratio 2:3) under stirring at room temperature until a clear and homogeneous solution was obtained.

*Secondary Polymerization Syrup (SPS) Formulation:* In separate amber glass vials, 1 g of the as–prepared PPS was mixed with 4 g of the LE. The designated crosslinker (MBA, DPE, or PDDA, 0.1 g) and additional photo–initiator BDK (0.01 g) were subsequently added. The mixture was stirred thoroughly until full dissolution and uniform dispersion were achieved.

#### 3.3.3. Film Casting and Final UV Curing

The resulting SPS was uniformly applied to both sides of a commercial polypropylene (PP) separator using a doctor blade. The coated separator was immediately irradiated under a 365 nm UV lamp for 10 min to induce complete curing. The resulting solid polymer electrolyte membranes (MFE, DFE, and PFE) were then cut into desired sizes and stored in an argon–filled glovebox for subsequent electrochemical characterization.

### 3.4. Electrode Preparation

Cathodes (Coin Cells): Cathode slurries were prepared by dispersing the active material (LFP or NCM622), Super P conductive carbon, and PVDF binder in NMP solvent at a mass ratio of 8:1:1. The mixture was stirred for at least 6 h and then uniformly cast onto carbon–coated aluminum foil (C–Al). The coated electrodes were dried at 80 °C, calendered, punched into 8 mm diameter disks, and subsequently vacuum–dried at 80 °C for no less than 12 h. The typical areal mass loading was 1.2–3.5 mg·cm^−2^, while higher loading (~8 mg·cm^−2^) was applied for high–voltage testing (e.g., under 4.5 V).

Cathodes (Pouch Cells): Single–crystal NCM811 (94.5 wt%), conductive agents, and binder were mixed and coated onto Al foil, then calendered. The areal capacity was ~1.63 mAh·cm^−2^, with dimensions of 50 mm × 50 mm.

Anodes (Coin Cells): Lithium metal foil disks with a diameter of 10 mm and a thickness of 0.5 mm were used as the anodes. For high–voltage tests (e.g., under 4.5 V), thinner lithium metal foils (10 mm diameter, 80 µm thickness) were employed.

Anodes (Pouch Cells): Graphite (Gr, 95.7 wt%), conductive agents, and binder were mixed and coated onto Cu foil, then calendered. The areal capacity was ~1.89 mAh·cm^−2^, with dimensions of 50 mm × 40 mm.

### 3.5. Electrochemical Characterization

The electrochemical properties were evaluated using Autolab Electrochemical Instrumentation (Metrohm, Herisau, Switzerland) or an equivalent potentiostat/galvanostat, primarily at 27 °C unless specified otherwise.

Ionic Conductivity (σ): This was evaluated using electrochemical impedance spectroscopy (EIS) on SS|PEs|SS cells over a frequency range from 100 kHz to 0.1 Hz. σ was calculated using the equation $\sigma =\frac{L}{RS}$, where L is the PEs membrane thickness, R is the bulk resistance derived from the Nyquist plot, and S is the electrode–electrolyte contact area.

Li^+^ Transference Number ($t_{{Li}^{+}}$): Determined using a combination of DC polarization and EIS on Li|PEs|Li symmetric cells. A voltage pulse (ΔV = 10 mV) was applied, and $t_{{Li}^{+}}$ was calculated via the Bruce–Vincent equation, $t_{{Li}^{+}}=\frac{I^{S}\left(\Delta V-I^{0}R^{0}\right)}{I^{0}\left(\Delta V-I^{S}R^{S}\right)}$, where $I^{0}$ and $I^{S}$ are the initial and steady–state currents, and $R^{0}$ and $R^{S}$ are the interfacial resistances before and after polarization, respectively.

Electrochemical Stability Window: This was assessed by linear sweep voltammetry (LSV) using SS|PEs|Li cells.

Interfacial Stability: This was evaluated by the galvanostatic cycling of Li|PEs|Li symmetric cells.

Cell Performance: The galvanostatic charge/discharge cycling of cells (LFP|PEs|Li, NCM622|PEs|Li, NCM811|PEs|Gr) was performed using a LAND–CT3001A battery tester (Wuhan LAND Electronic Co., Ltd., Wuhan, China) or equivalent. LiFePO_4_ cells were typically tested within a voltage range of 2.5–4.0 V vs. Li/Li^+^.

## 4. Conclusions

In summary, this study demonstrates enhanced ion transport in polymer electrolytes achieved by regulating the Li^+^ solvation structure via engineered hydrogen bond networks using an MBA crosslinker. Validated by computational modeling, this strategy effectively promotes salt dissociation and creates efficient Li^+^–conductive pathways. The resultant MFE electrolyte manifested superior ionic conductivity (5.1 × 10^−4^ S cm^−1^) coupled with a substantially enhanced Li^+^ transference number (t_Li+_ = 0.57). These synergistic transport characteristics facilitated exceptional electrochemical stability across multiple configurations, evidenced by the stable cycling of Li|MFE|Li symmetric cells exceeding 4000 h; remarkable long–term stability in LFP|MFE|Li cells, which maintained >81% capacity retention after 1400 cycles at 1C; and outstanding performance in NCM622|MFE|Li cells, preserving > 97% of their initial capacity following 800 cycles at 0.5C. Stable Li interfaces and successful pouch cell operation confirm the proposed strategy’s practical viability. This work highlights synergistic hydrogen bonding as a potent molecular engineering approach to tailor solvation environments, paving a promising path towards high–performance solid–state lithium metal batteries.

## Data Availability

The datasets generated and/or analyzed during the current study are available from the corresponding authors upon reasonable request.

## Supplementary Materials

The following supporting information can be downloaded at: https://www.mdpi.com/article/10.3390/molecules30112474/s1, Figure S1. (a–h) RDF and CN of Li^+^ with polymers, anions, crosslinkers, solvents, and Li^+^ in MFE, DFE, and PFE; Figure S2. Mean square displacement (MSD) results of Li^+^ in MFE, DFE, and PFE polymer electrolytes derived from MD simulations; Figure S3. (a–d) Spatial density profiles of Li^+^, O (ester), S, and B; Figure S4. LSV curves of SS|PEs|Li cells scanned from 3.0 V to 6.0 V; Figure S5. Cyclic voltammetry curves of Li|Cu half cells used to evaluate the reversibility of lithium plating and stripping in different polymer electrolyte systems; Figure S6. (a–c) Voltage profile evolution of LFP|Li full cells cycled at the 1C rate over different cycle numbers for the three polymer electrolyte systems (MFE, DFE, and PFE); Figure S7. Initial interfacial impedance spectra of LFP|Li full cells assembled with MFE, DFE, and PFE PEs; Figure S8. (a–c) Voltage profile evolution of NCM622|Li full cells cycled at the 0.5C rate over different cycle numbers for the three polymer electrolyte systems (MFE, DFE, and PFE); Figure S9. Comparison of σ in different electrolytes; Figure S10. (a–c) Comparison of t_Li+_ in different electrolytes; Figure S11. (a–c) AFM height images of the polymer electrolyte membranes MFE, DFE, and PFE. These topographic maps reveal nanoscale surface morphology and roughness differences among the three samples. The corresponding root–mean–square roughness (R_q_) and average roughness (R_a_) values are provided below each image; Figure S12. (a–c) Quantitative nanomechanical mapping (QNM) images obtained via AFM, showing the spatial distribution of Young’s modulus (E_Y_) across the surface of the MFE, DFE, and PFE polymer electrolyte membranes; Figure S13. Stress–strain curves; Figure S14. Cycling performance of LFP|Gr pouch cell using MFE; Figure S15. FTIR spectra of HFMA monomer and partially polymerized HFMA (pHFMA) obtained from the first–stage UV photo–polymerization process; Table S1. Electrochemical performance of the MFE–based PEs (this work) compared with previously reported polymer electrolyte systems; Table S2. Raw data used for the determination of Li^+^ transference number (t_Li_^+^); Table S3. Molar ratio composition of raw materials in the resulting polymer electrolytes (PEs). References [30,31,32,33,34,35] are cited in the Supplementary Materials.

## References

[1] Xia S., Wu X., Zhang Z., Cui Y., Liu W. (2019). Practical Challenges and Future Perspectives of All–Solid–State Lithium–Metal Batteries. Chem.

[2] Gao X.-Z., Yuan W., Yang Y., Wu Y.-P., Wang C., Wu X.-Y., Zhang X.-Q., Yuan Y.-H., Tang Y., Chen Y. (2022). High–Performance and Highly Safe Solvate Ionic Liquid–Based Gel Polymer Electrolyte by Rapid UV–Curing for Lithium–Ion Batteries. ACS Appl. Mater. Interfaces.

[3] Tseng Y.-C., Hsiang S.-H., Lee T.-Y., Teng H.-S., Jan J.-S., Thein K. (2021). In–situ Polymerized Electrolytes with Fully Cross–Linked Networks Boosting High Ionic Conductivity and Capacity Retention for Lithium Ion Batteries. ACS Appl. Energy Mater..

[4] Feng Y., Zhou L., Ma H., Wu Z., Zhao Q., Li H., Zhang K., Chen J. (2022). Challenges and Advances in Wide–Temperature Rechargeable Lithium Batteries. Energy Environ. Sci..

[5] Oh P., Lee H., Park S., Cha H., Kim J., Cho J. (2020). Improvements to the Overpotential of All–Solid–State Lithium–Ion Batteries during the Past Ten Years. Adv. Energy Mater..

[6] Sashmitha K., Rani M.U. (2023). A Comprehensive Review of Polymer Electrolyte for Lithium–Ion Battery. Polym. Bull..

[7] Yang X., Jiang M., Gao X., Bao D., Sun Q., Holmes N., Duan H., Mukherjee S., Adair K., Zhao C. (2020). Determining the Limiting Factor of the Electrochemical Stability Window for PEO–Based Solid Polymer Electrolytes: Main Chain or Terminal –OH Group?. Energy Environ. Sci..

[8] Zhou D., Shanmukaraj D., Tkacheva A., Armand M., Wang G. (2019). Polymer Electrolytes for Lithium–Based Batteries: Advances and Prospects. Chem.

[9] Kumar R., Sharma J.P., Sekhon S. (2005). FTIR Study of Ion Dissociation in PMMA Based Gel Electrolytes Containing Ammonium Triflate: Role of Dielectric Constant of Solvent. Eur. Polym. J..

[10] Yao N., Chen X., Shen X., Zhang R., Fu Z.-H., Ma X.-X., Zhang X.-Q., Li B.-Q., Zhang Q. (2021). An Atomic Insight into the Chemical Origin and Variation of the Dielectric Constant in Liquid Electrolytes. Angew. Chem. Int. Ed..

[11] Guo C., Du K., Tao R., Guo Y., Yao S., Wang J., Wang D., Liang J., Lu S.Y. (2023). Inorganic Filler Enhanced Formation of Stable Inorganic–Rich Solid Electrolyte Interphase for High Performance Lithium Metal Batteries. Adv. Funct. Mater..

[12] Cui J., Liu Y., Du L., Zhao P., Wang D., Li S., Zhang Y., Li H. (2025). Self–Regulating the Local Conjugation of Tertiary Aniline toward Highly Stable Polymer Li Metal Batteries. Adv. Mater..

[13] Zhou W., Wang Z., Pu Y., Li Y., Xin S., Li X., Chen J., Goodenough J.B. (2019). Double–Layer Polymer Electrolyte for High–Voltage All–Solid–State Rechargeable Batteries. Adv. Mater..

[14] Wang P., Chai J., Zhang Z., Zhang H., Ma Y., Xu G., Du H., Liu T., Li G., Cui G. (2019). An Intricately Designed Poly(vinylene carbonate–acrylonitrile) Copolymer Electrolyte Enables 5 V Lithium Batteries. J. Mater. Chem. A.

[15] Imholt L., Dong D., Bedrov D., Cekic-Laskovic I., Winter M., Brunklaus G. (2018). Supramolecular Self–Assembly of Methylated Rotaxanes for Solid Polymer Electrolyte Application. ACS Macro Lett..

[16] Park C.H., Kim D.W., Prakash J., Sun Y.-K. (2003). Electrochemical Stability and Conductivity Enhancement of Composite Polymer Electrolytes. Solid State Ionics.

[17] Liu W., Lin D., Sun J., Zhou G., Cui Y. (2016). Improved Lithium Ionic Conductivity in Composite Polymer Electrolytes with Oxide–Ion Conducting Nanowires. ACS Nano.

[18] Pei F., Wu L., Zhang Y., Liao Y., Kang Q., Han Y., Zhang H., Shen Y., Xu H., Li Z. (2023). Interfacial Self–Healing Polymer Electrolytes for Ultralong–Life Solid–State Lithium–Sulfur Batteries. Nat. Commun..

[19] Chen H.W., Jiang C.H., Wu H.D., Chang F.C. (2004). Hydrogen Bonding Effect on the Poly(ethylene oxide), Phenolic Resin, and Lithium Perchlorate–Based Solid–State Electrolyte. J. Appl. Polym. Sci..

[20] Cheng C.-C., Lee D.-J. (2016). Supramolecular Assembly–Mediated Lithium Ion Transport in Nanostructured Solid Electrolytes. RSC Adv..

[21] Lv Z., Tang Y., Dong S., Zhou Q., Cui G. (2022). Polyurethane–Based Polymer Electrolytes for Lithium Batteries: Advances and Perspectives. Chem. Eng. J..

[22] Huang Y.J., Shi Z., Wang H.L., Wang J.R., Xue Z.G. (2022). Polyethylene Glycol–Based Waterborne Polyurethane as Solid Polymer Electrolyte for All–Solid–State Lithium Ion Batteries. Energy Storage Mater..

[23] Qi S., Li M., Gao Y., Zhang W., Liu S., Zhao J., Du L. (2023). Enabling Scalable Polymer Electrolyte with Dual–Reinforced Stable Interface for 4.5 V Lithium Metal Batteries. Adv. Mater..

[24] Gao Y., Qi S., Li M., Ma T., Song H., Cui Z., Liang Z., Du L. (2024). Machine Learning Assisted Prediction of Donor Numbers: Guiding Rational Fabrication of Polymer Electrolytes for Lithium–Ion Batteries. Angew. Chem. Int. Ed..

[25] Rajahmundry G.K., Patra T.K. (2024). Understanding Ion Distribution and Diffusion in Solid Polymer Electrolytes. Langmuir.

[26] Choo Y., Halat D.M., Villaluenga I., Timachova K., Balsara N.P. (2020). Diffusion and Migration in Polymer Electrolytes. Prog. Polym. Sci..

[27] Röchow E.T., Coeler M., Pospiech D., Kobsch O., Mechtaeva E., Vogel R., Voit B., Nikolowski K., Wolter M. (2020). In Situ Preparation of Crosslinked Polymer Electrolytes for Lithium Ion Batteries: A Comparison of Monomer Systems. Polymers.

[28] Xu A.H., Zhang L.Q., Ma J.C., Ma Y.M., Geng B., Zhang S.X. (2016). Preparation and Surface Properties of Poly(2,2,2–trifluoroethyl Methacrylate) Coatings Modified with Methyl Acrylate. J. Coat. Technol. Res..

[29] Qi S.G., Li S.L., Zou W.W., Zhang W.F., Wang X.J., Du L., Liu S.M., Zhao J.Q. (2022). Enabling Scalable Polymer Electrolyte with Synergetic Ion Conductive Channels via a Two Stage Rheology Tuning UV Polymerization Strategy. Small.

[30] Li Y., Sun Z., Liu D., Gao Y., Wang Y., Bu H., Li M., Zhang Y., Gao G., Ding S. (2020). A Composite Solid Polymer Electrolyte Incorporating MnO_2_ Nanosheets with Reinforced Mechanical Properties and Electrochemical Stability for Lithium Metal Batteries. J. Mater. Chem. A.

[31] Pei D., Ma R., Yang G., Li Y., Huang C., Liu Z., Huang S., Cao G., Jin H. (2022). Enhanced Ion Transport Behaviors in Composite Polymer Electrolyte: The Case of a Looser Chain Folding Structure. J. Mater. Chem. A.

[32] Atik J., Diddens D., Thienenkamp J.H., Brunklaus G., Winter M., Paillard E. (2021). Cation–Assisted Lithium–Ion Transport for High–Performance PEO–Based Ternary Solid Polymer Electrolytes. Angew. Chem. Int. Ed..

[33] Yuan B., Luo G., Liang J., Cheng F., Zhang W., Chen J. (2019). Self–Assembly Synthesis of Solid Polymer Electrolyte with Carbonate Terminated Poly(ethylene glycol) Matrix and Its Application for Solid–State Lithium Bakery. J. Energy Chem..

[34] Li H., Du Y., Wu X., Xie J., Lian F. (2021). Developing “Polymer–in–Salt” High Voltage Electrolyte Based on Composite Lithium Salts for Solid–State Li Metal Bakeries. Adv. Funct. Mater..

[35] Dong T., Zhang H., Hu R., Mu P., Liu Z., Du X., Lu C., Lu G., Liu W., Cui G. (2022). A Rigid–Flexible Coupling Poly(vinylene carbonate)–Based Cross–Linked Network: A Versatile Polymer Platform for Solid–State Polymer Lithium Bakeries. Energy Storage Mater..

