# Supplementary material for: Enhancing Ion Transport in Polymer Electrolytes by Regulating Solvation Structure via Hydrogen Bond Networks
Source: Molecules. 2025 Jun 5;30(11):2474. doi: 10.3390/molecules30112474 (PMC12156010; doi:10.3390/molecules30112474)
Supplement: Supplementary file 1 [file molecules-30-02474-s001.zip › molecules-3614844-supplementary.pdf]

Supporting Information

Enhancing Ion Transport in Polymer Electrolytes by Regulating Solvation Structure via Hydrogen Bond Networks

Yuqing Gao †, Yankui Mo †, Shengguang Qi, Mianrui Li, Tongmei Ma and Li Du *

Guangdong Provincial Key Laboratory of Fuel Cell Technology, School of Chemistry and Chemical Engineering, South China University of Technology, Guangzhou 510640, China
* Correspondence: duli@scut.edu.cn
† These authors contributed equally to this work.

2. Additional Data

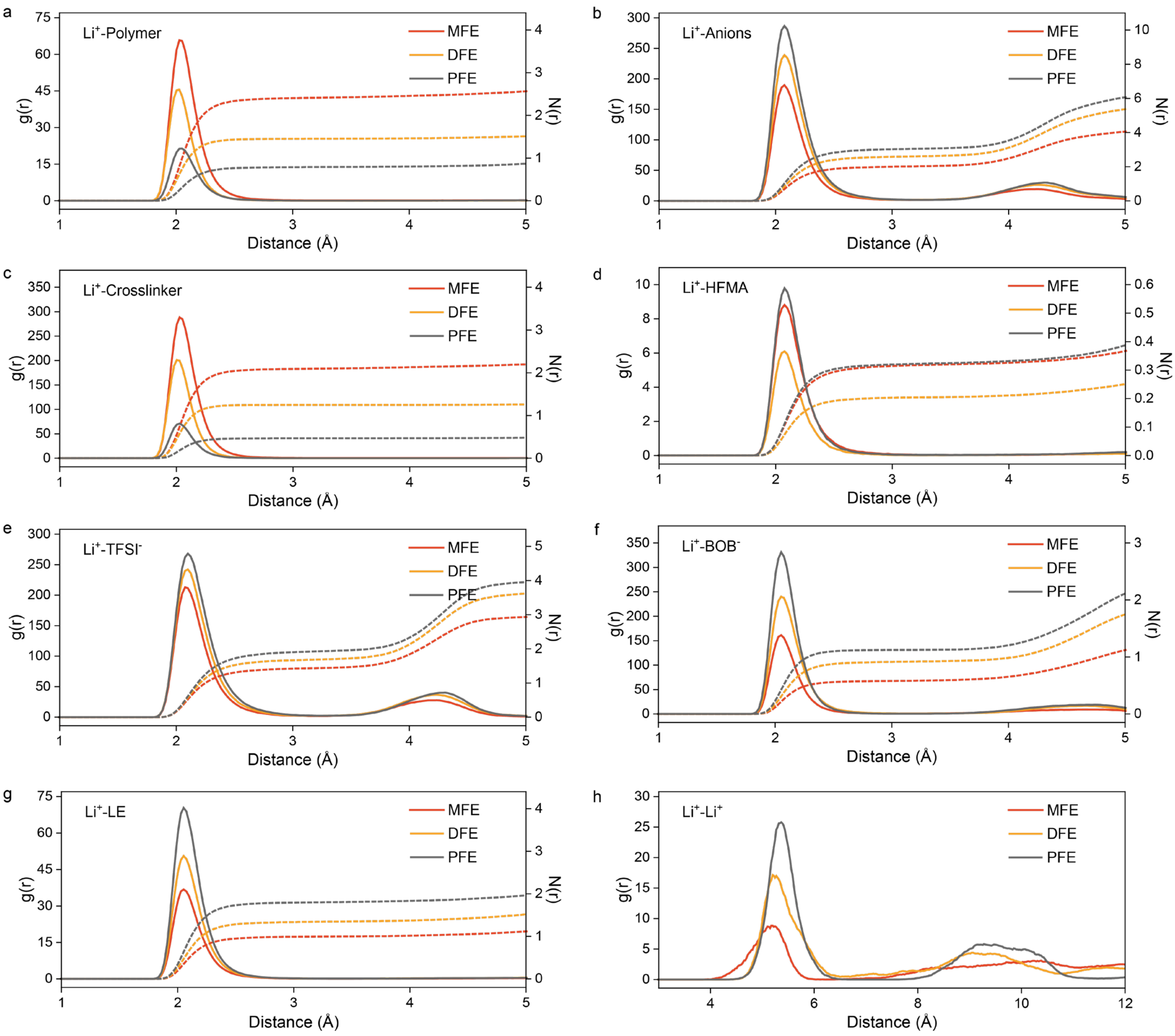

Figure S1 (a–h) RDF and CN of Li^+ with polymers, anions, crosslinkers, solvents, and Li^+ in MFE, DFE, and PFE.

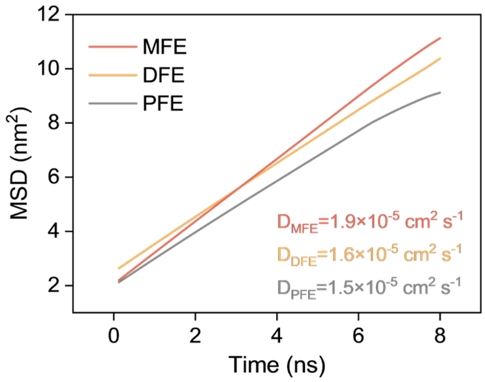

Figure S2. Mean square displacement (MSD) results of Li^+ in MFE, DFE, and PFE polymer electrolytes derived from MD simulations.

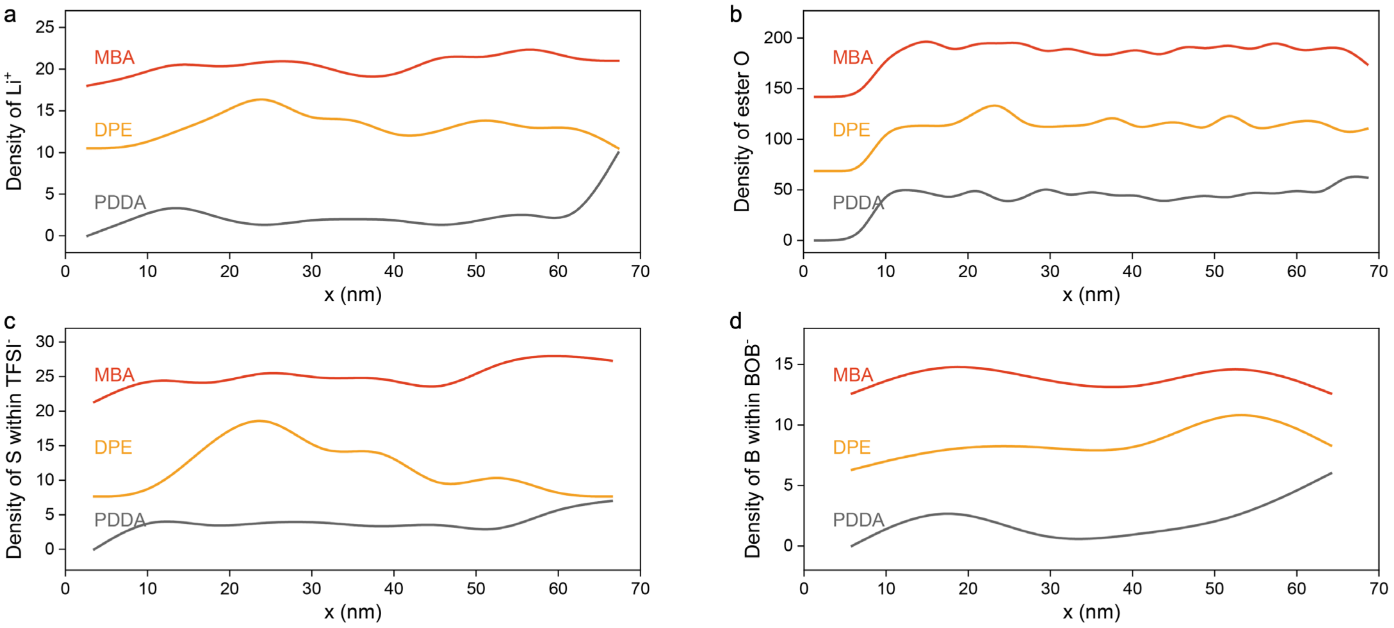

Figure S3. (a–d) Spatial density profiles of Li^+, O (ester), S, and B.

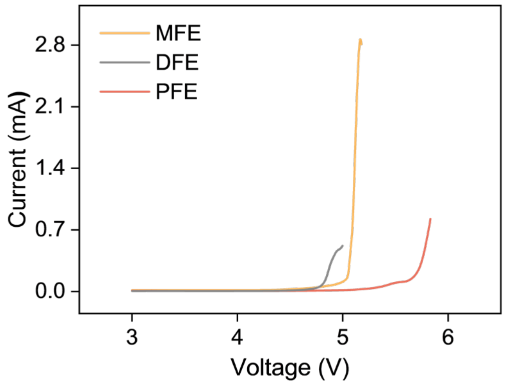

Figure S4. LSV curves of SS|PEs|Li cells scanned from 3.0 V to 6.0 V.

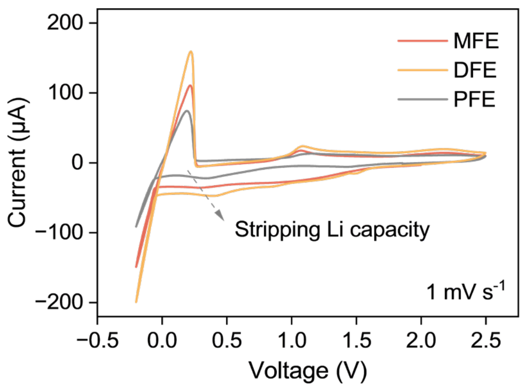

Figure S5. Cyclic voltammetry curves of Li|Cu half cells used to evaluate the reversibility of lithium plating and stripping in different polymer electrolyte systems.

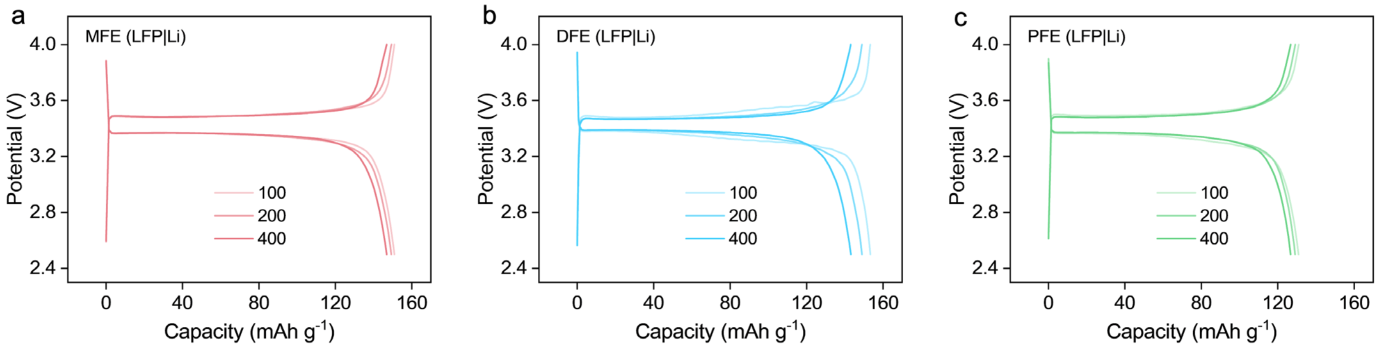

Figure S6. (a–c) Voltage profile evolution of LFP|Li full cells cycled at the 1C rate over different cycle numbers for the three polymer electrolyte systems (MFE, DFE, and PFE).

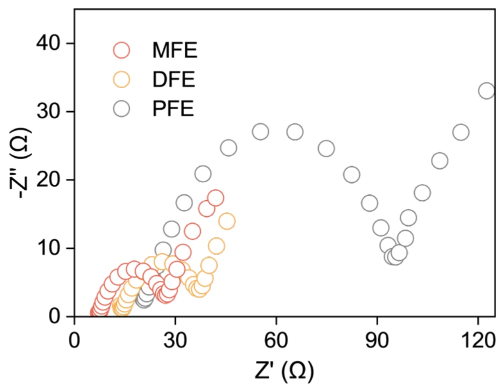

Figure S7. Initial interfacial impedance spectra of LFP|Li full cells assembled with MFE, DFE, and PFE PEs.

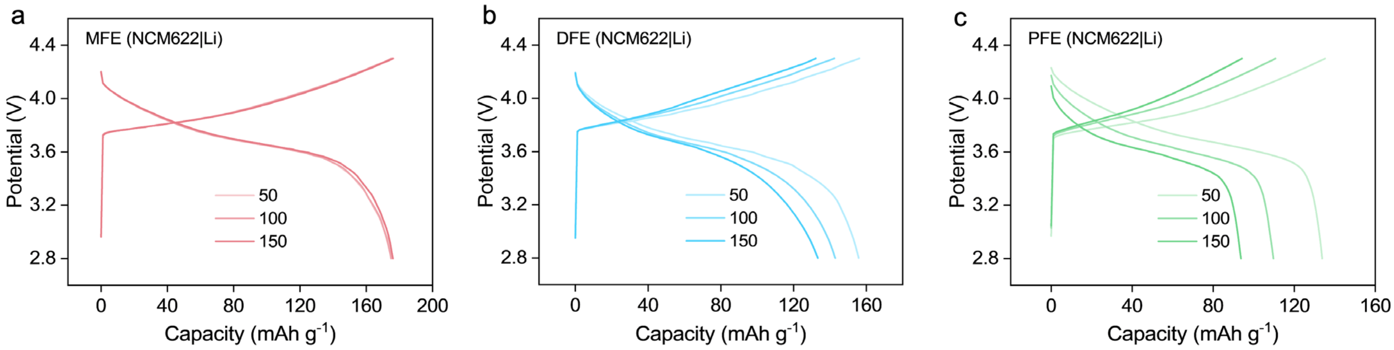

Figure S8. (a–c) Voltage profile evolution of NCM622|Li full cells cycled at the 0.5C rate over different cycle numbers for the three polymer electrolyte systems (MFE, DFE, and PFE).

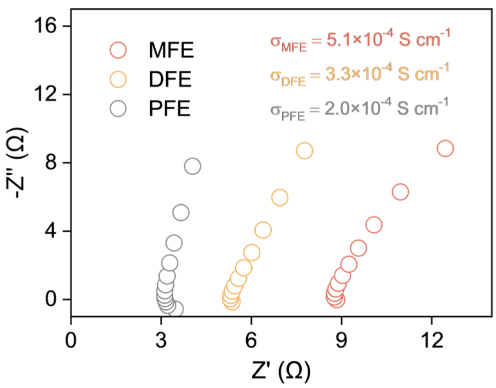

Figure S9. Comparison of σ in different electrolytes.

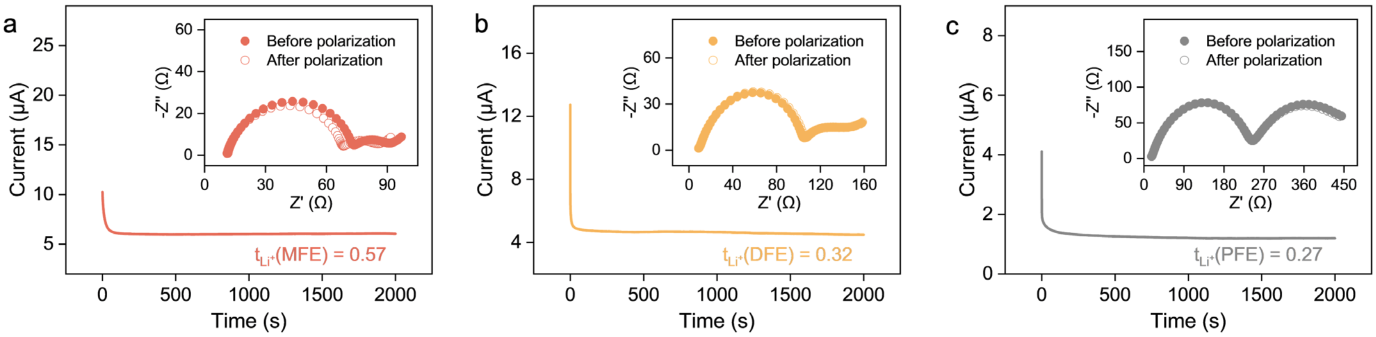

Figure S10. (a–c) Comparison of t_{Li+} in different electrolytes.

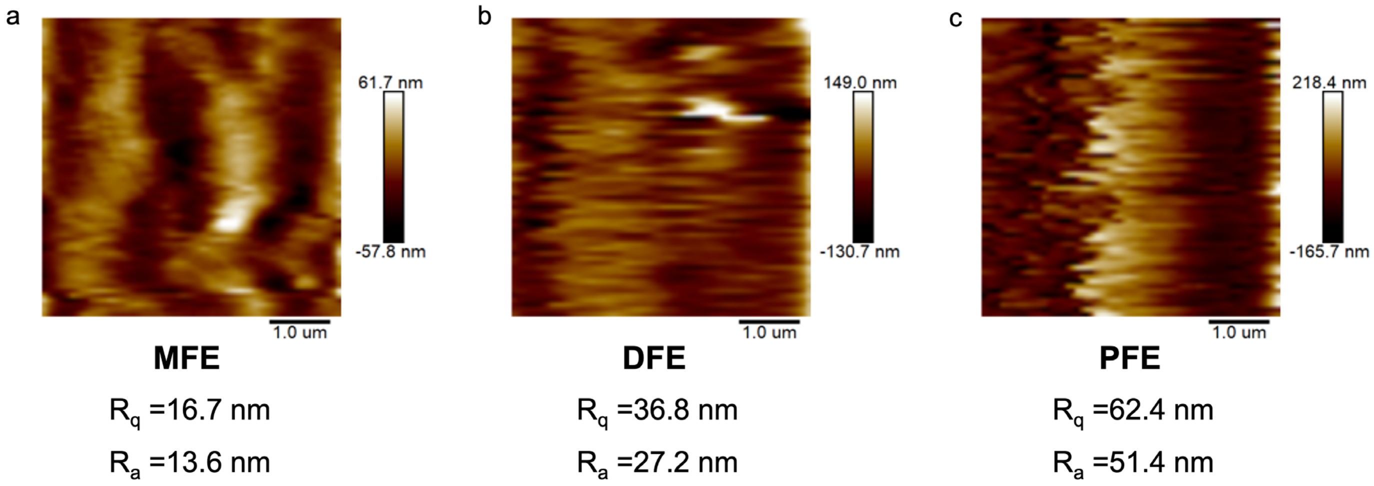

Figure S11. (a–c) AFM height images of the polymer electrolyte membranes MFE, DFE, and PFE. These topographic maps reveal nanoscale surface morphology and roughness differences among the three samples. The corresponding root–mean–square roughness (R_q) and average roughness (R_a) values are provided below each image.

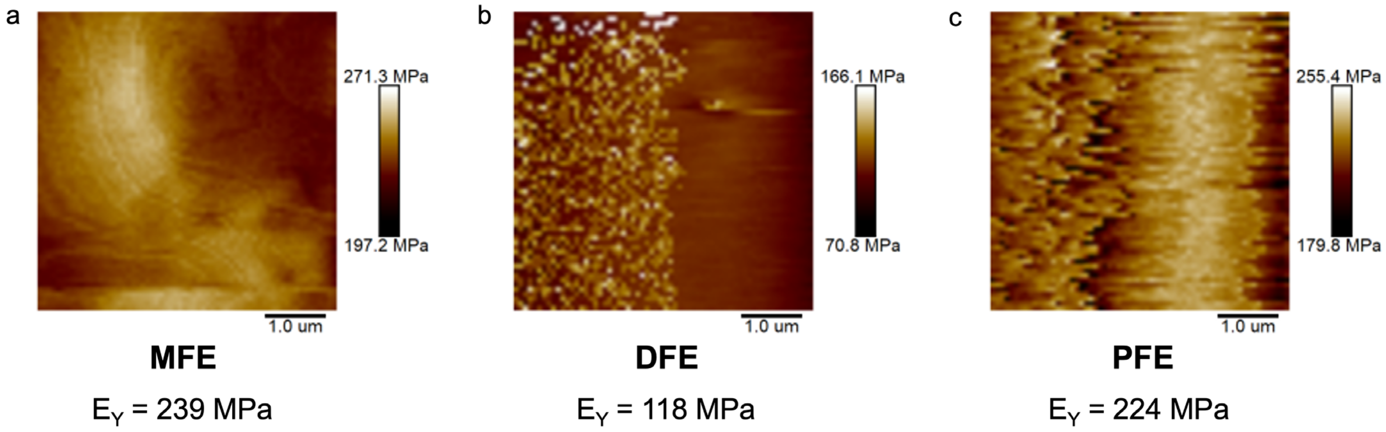

Figure S12. (a–c) Quantitative nanomechanical mapping (QNM) images obtained via AFM, showing the spatial distribution of Young's modulus (E_Y) across the surface of the MFE, DFE, and PFE polymer electrolyte membranes.

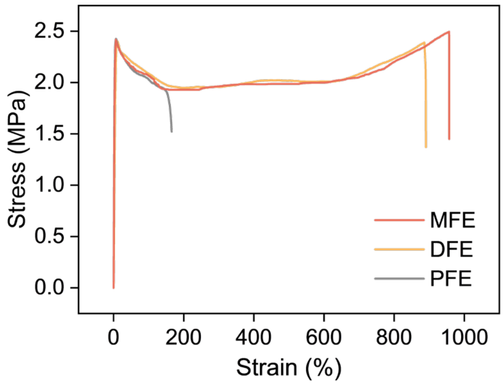

Figure S13. Stress–strain curves.

As illustrated in Figure S13, the MFE membrane exhibited the most desirable mechanical behavior, with a high tensile strength and an extended strain plateau, indicative of both rigidity and ductility. In contrast, the PFE membrane demonstrated premature stress drop and unstable deformation, suggesting a less interconnected polymer network. The DFE membrane showed intermediate performance, reflecting a dense but relatively brittle structure. These results highlight the crucial role of hydrogen bond−regulated solvation structures in tuning the mechanical resilience of polymer electrolytes, which is essential not only for maintaining dimensional stability but also for ensuring reliable interfacial contact and mitigating lithium dendrite formation in battery operation.

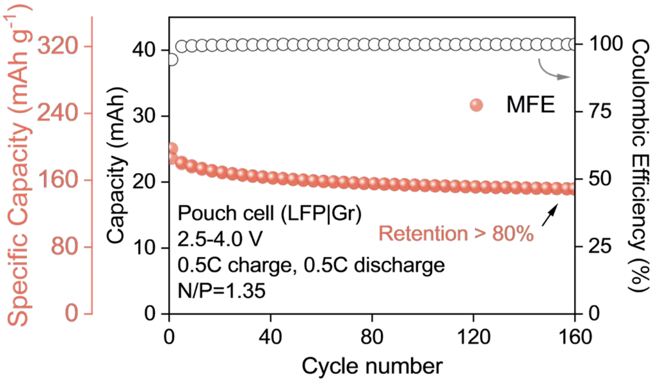

Figure S14. Cycling performance of LFP|Gr pouch cell using MFE.

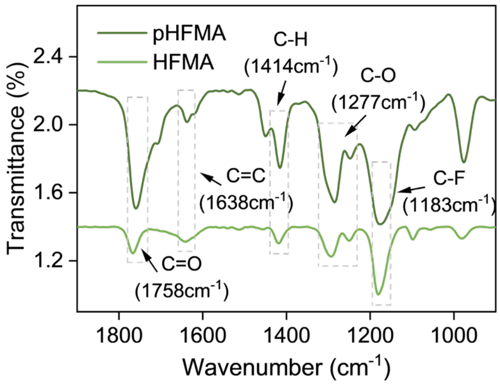

Figure S15. FTIR spectra of HFMA monomer and partially polymerized HFMA (pHFMA) obtained from the first−stage UV photopolymerization process.

Table S1 Electrochemical performance of the MFE–based PEs (this work) compared with previously reported polymer electrolyte systems.

PEs	Operating temperature (℃)	σ (mS cm^{-1})	t_{Li+}	Thicknes (μm)	Cathode	Retention rate /Cycle number/C rate	Ref.
**MFE**	**27**	**0.51**	**0.57**	**18**	**LFP**	**82%/1400/3C**	**This work**
					**NCM622**	**81%/500/1C**	
$PEO/LiTFSI/MnO_2$	60	0.02	0.38	90	LFP	86.7%/300/0.5 C	[1]
$PCL/LiTFSI/Al_2O_3$	60	0.05	0.65	–	LFP	81.3%/500/1.0 C	[2]
PEO/LiTFSI/IL	40	0.66	0.10	–	LFP	99.3%/200/1 C	[3]
$PEG/LiTFSI/SiO_2$	26	0.17	0.53	–	LFP	68.1%/300/0.2 C	[4]
PEGDE–PEA	30	0.70	0.47	～30	LCO	91.95%/150/ 0.2 C	[5]
PVC/POEA/AN	60	0.045	0.32	–	LCO	80%/150/0.5 C	[6]

Table S2 Raw data used for the determination of Li^+ transference number (t_{Li^+}).

PEs	I^0 (μA)	I^s (μA)	R^0 (Ω)	R^s (Ω)	△V (mV)	t_{Li+}
MFE	10.24	6.05	73.43	68.33	10	0.57
DFE	12.72	4.48	106.45	106.88	10	0.32
PFE	4.11	1.20	243.61	245.42	10	0.27

Table S3 Molar ratio composition of raw materials in the resulting polymer electrolytes (PEs).

PEs	EC	EMC	LITFSI	LIBOB	Crosslinker	HFMA	BDK
MFE	17.28	16.81	1.74	1.16	0.65	5.95	0.04
DFE	17.28	16.81	1.74	1.16	0.51	5.95	0.04
PFE	17.28	16.81	1.74	1.16	0.33	5.95	0.04

References

1. Li, Y.; Sun, Z.; Liu, D.; Gao, Y.; Wang, Y.; Bu, H.; Li, M.; Zhang, Y.; Gao, G.; Ding, S. A Composite Solid Polymer Electrolyte Incorporating MnO_2 Nanosheets with Reinforced Mechanical Properties and Electrochemical Stability for Lithium Metal Batteries. *J. Mater. Chem. A* **2020**, *8*, 2021.
2. Pei, D.; Ma, R.; Yang, G.; Li, Y.; Huang, C.; Liu, Z.; Huang, S.; Cao, G.; Jin, H. Enhanced Ion Transport Behaviors in Composite Polymer Electrolyte: The Case of a Looser Chain Folding Structure. *J. Mater. Chem. A* **2022**, *10*, 3226.
3. Atik, J.; Diddens, D.; Thienenkamp, J. H.; Brunklaus, G.; Winter, M.; Paillard, E. Cation−Assisted Lithium−Ion Transport for High−Performance PEO−Based Ternary Solid Polymer Electrolytes. *Angew. Chem. Int. Ed.* **2021**, *60*, 11919.
4. Yuan, B.; Luo, G.; Liang, J.; Cheng, F.; Zhang, W.; Chen, J. Self−Assembly Synthesis of Solid Polymer Electrolyte with Carbonate Terminated Poly(ethylene glycol) Matrix and Its Application for Solid−State Lithium Battery. *J. Energy Chem.* **2019**, *38*, 55.
5. Li, H.; Du, Y.; Wu, X.; Xie, J.; Lian, F. Developing “Polymer−in−Salt” High Voltage Electrolyte Based on Composite Lithium Salts for Solid−State Li Metal Batteries. *Adv. Funct. Mater.* **2021**, *31*, 2103049.
6. Dong, T.; Zhang, H.; Hu, R.; Mu, P.; Liu, Z.; Du, X.; Lu, C.; Lu, G.; Liu, W.; Cui, G. A Rigid−Flexible Coupling Poly(vinylene carbonate)−Based Cross−Linked Network: A Versatile Polymer Platform for Solid−State Polymer Lithium Batteries. *Energy Storage Mater.* **2022**, *50*, 525.
